# Exploring sustainable leadership among first-line managers in healthcare: a qualitative study

**DOI:** 10.1186/s12913-026-14412-6

**Published:** 2026-03-23

**Authors:** Anna Marzelius, Petra Svedberg, Jens Nygren, Björn Agvall, Anna Gyberg, Ingrid Larsson

**Affiliations:** 1 School of Health and Welfare, Halmstad University, P.O. Box 823, Halmstad, S-30118 Sweden; 2 Department of Surgery and Intensive Care, Hospital of Halland, Region Halland, Varberg, Sweden; 3 Department of Research and Development, Region Halland, Halmstad, Sweden; 4 Center for Primary Health Care Research, Department of Clinical Sciences, Lund University, Malmö, Sweden

**Keywords:** Healthcare managers, Job satisfaction, Leadership, Organizational culture, Sustainable development, Work-life balance

## Abstract

**Background:**

First-line managers in healthcare operate in increasingly complex environments marked by high demands, rapid change, and limited resources. These conditions impact their health, leadership capacity, and the sustainability of their roles. While previous research has addressed various aspects of leadership in healthcare, there is a lack of studies focusing specifically on how first-line managers perceive the conditions for sustainable leadership in a changing and resource-demanding organizational context. The aim of this study was to explore the conditions for sustainable leadership among first-line managers in healthcare settings.

**Method:**

Using an exploratory qualitative design with an inductive approach, individual interviews were conducted with 25 first-line managers from diverse healthcare areas in southern Sweden between February and July 2024. Three main themes, and nine sub-themes related to sustainable leadership emerged from the qualitative content analysis.

**Results:**

Providing resilient organizational structures was characterised by support functions that met operational requirements, adequate resource allocation, and coordinated work routines, enabling managers to carry out leadership tasks over time. Fostering supportive collaborations comprised supervisory and peer support, opportunities for collegial exchange, and access to administrative assistance. These forms of support affected how managers handled their responsibilities and responded to organizational change. Cultivating leadership for innovation included linking leadership tasks to a clear purpose, managing changing expectations, and balancing operational duties with broader development goals.

**Conclusion:**

Sustainable leadership among first-line managers depends on the alignment of structural, social, and individual conditions. The findings indicate that resilient organizational systems, accessible support networks, and possibilities to integrate purpose with daily leadership are crucial for maintaining leadership capacity over time. These insights can guide organizational strategies aimed at strengthening managers’ prerequisites for sustainable leadership in complex healthcare settings.

**Supplementary Information:**

The online version contains supplementary material available at 10.1186/s12913-026-14412-6.

## Introduction

The changing healthcare environment is increasingly shaped by rapid digitalization and the introduction of advanced technologies such as artificial intelligence (AI), which significantly alter the conditions under which care is delivered and managed [1]. These changes place new and complex demands on managers, who are expected to broaden their digital skills, strengthen their communicative leadership, and foster innovation [2]. Research has shown that digital leadership skills are positively associated with psychological well-being among managers [3]. At the same time, first-line managers’ experiences of stress, high workload, and lack of organizational support may undermine their ability to sustain leadership over time and increase the risk of leaving the role [4, 5]. Trust, responsibility, and opportunities to make a meaningful difference have, conversely, been described as important for remaining in the role [5]. Previous research has primarily focused on how leadership is enacted and on conditions described as important for first-line managers’ retention, such as trust, autonomy, collegial support, and meaningful work [5], or linked to turnover risk, such as low control and limited manager support [6]. Less attention has been paid to the broader conditions that enable first-line managers to sustain their leadership over time under increasing complexity and change. In this study, a sustainable leadership perspective is therefore adopted. Sustainable leadership refers to first-line managers’ capacity to maintain leadership over time in contexts of ongoing organizational change, particularly digitalization and AI implementation [1, 2]. Rather than focusing on leadership outcomes or environmental sustainability, the concept concerns the endurance and continuity of managers’ ability to perform leadership in everyday practice. This includes managing complexity and workload [4, 7],while also having access to organizational and relational conditions such as trust, managerial and collegial support over time [5].

First-line managers in healthcare organizations, who are directly responsible for staff and daily care, play a critical role by translating strategic goals into practice, ensuring high-quality patient care, and promoting team performance [8, 9]. Their proximity to frontline staff and patients positions them as key actors in implementing improvements and innovations in care delivery. Sustainable leadership in times of digital transformation at this level is essential not only for fostering a supportive and quality-driven work environment but also for promoting employee well-being and the long-term resilience of healthcare services [3, 10]. Insufficient support and high work demands have been shown to increase the risk of managerial turnover, particularly when control and managerial support are perceived as inadequate [5, 6].

Despite the importance of first-line managers’ responsibilities in healthcare, they often work under challenging conditions characterized by high workloads, limited resources, and increasing demands for efficiency and quality [10, 11]. Such pressures can result in emotional exhaustion and burnout, ultimately compromising first-line managers’ leadership capacity and reducing their ability to effectively support staff and maintain a healthy work environment. Furthermore, when managers at different levels feel insufficiently recognized or rewarded for their efforts, the risk of burnout increases [11, 12], which may further undermine their leadership capacity and ability to provide security and support for employees [9].

First-line managers’ capacity to cope with stress and lead sustainably is shaped by a dynamic interplay of individual, organizational [8, 13, 14], and relational conditions [4, 5]. At the individual level, personal values, experiences, and individual resources may strengthen leadership capacity [10]. At the organizational level, clearly defined goals, manageable workloads, access to resources and support functions [10, 13], and opportunities for recovery are essential for preventing stress-related strain and enabling sustainable managerial work [8, 15]. At the relational level, trust, collegial support, and support from one’s immediate manager appear important for sustaining leadership over time [4, 5]. Supporting these conditions is essential in today’s complex healthcare environment. When first-line managers are able to sustain their leadership over time [6, 8], this may not only protect their own health but also create conditions for a sustainable workforce in which employees remain motivated and engaged in delivering care [16, 17].

Despite growing interest in sustainable leadership [2, 8, 10], there is limited qualitative research on how first-line managers describe the conditions that make sustainable leadership possible in everyday practice, particularly in a Swedish healthcare context [8, 10, 15]. By focusing on first-line managers’ own experiences, this study adds context-specific knowledge about these conditions and can inform efforts to better support sustainable leadership [3, 8, 10, 15]. Therefore, the aim of this study was to explore the conditions for sustainable leadership among first-line managers in healthcare settings.

## Method

### Design

The study used an exploratory design with qualitative content analysis [18, 19] and an inductive approach to uncover similarities, differences, and variations in experiences. This approach was appropriate for capturing variations in how first-line managers experienced and described the conditions that support sustainable leadership. The study is reported in accordance with the consolidated criteria for reporting qualitative research (COREQ) 32-item checklist to ensure trustworthiness [20].

### Setting and participants

The study was conducted in a regional healthcare authority comprising three hospitals located at different sites in southern Sweden. First-line managers in this context are operational-level leaders with daily responsibility for core activities and direct supervision of clinically active staff groups, typically overseeing 35–40 healthcare professionals.

The inclusion criterion for participants was: being employed as a first-line manager with responsibility for clinical staff at one of the three hospitals. All first-line managers working at these hospitals in February 2023 (*N* = 137) were invited via e-mail to participate, aiming to achieve variation across departments and managerial experience. Seventeen of these accepted the invitation, five declined, and 115 did not respond. A second invitation was sent to the non-responders, resulting in eight additional participants. A total of 25 first-line managers from various hospital departments participated in the study. The sampling strategy enabled the inclusion of managers with experience across a broad range of clinical specialties, including general internal medicine, surgical specialties, anesthesiology and perioperative care, emergency, intensive care medicine, obstetrics, technical services, and staffing units. The participants consisted of 23 women and two men, with a median age of 46 years (range 35 to 63 years) and a median of 5.5 years of work experience as a first-line manager (range 0.5 to 21 years) (Table 1).

**Table 1:** Sociodemographic data of the participants (*n* = 25)

*Characteristics*	*Value*
Sex (female/male) (n)	23 / 2
Age (years), Median (range)	46 (35–63)
Workplace (n): - Inpatient Care - Outpatient Care - Inpatient and Outpatient Care - Service functions supporting in- and outpatient care**	16 2 4 3
Education Level (Secondary Level / University) (n)	2/23
Manager Education (n)	24
Experience as FLM* (years), Median (range)	5 (0.5–20)
Experience as FLM* from more than one hospital (n)	7

* Abbreviations: FLM: First-Line Manager, **For example, *Technical services*,* Staffing unit*

### Data collection

The data collection was conducted with individual interviews from March to July 2024. The interviews, including the interview guide, were developed specifically for this research project and have not been used or published previously.

Twenty-two interviews were conducted in person and took place in a quiet room, and three were conducted digitally based on participant preferences. The interviews were conducted by either the first (AM) or the last (IL) author.

The interviews were based on an interview guide with open-ended questions focusing on sustainable leadership and the ability to lead during change in order to allow participants to describe their experiences and perspectives. The three main questions posed were: What does sustainable leadership as a manager mean to you, and how would you describe it? Which opportunities do you have to achieve a sustainable working life, and how do you experience them? Which obstacles do you see in achieving a sustainable working life, and how do you perceive them? Probing questions were asked, such as ‘Can you tell me more about…,’ ‘What do you mean,’ ‘Can you describe that further,’ ‘Why,’ or ‘What did you have in mind when you said…?’ All interviews were audio-recorded and transcribed verbatim. They lasted between 36 and 111 min, with a total combined duration of 21.3 h. The average length was 51 min, with a median of 57 min.

### Data analysis

A qualitative content analysis was applied to enable the researcher to interpret the meaning of textual data, thereby allowing an understanding of the conditions surrounding the leadership of first-line managers [19]. A total of 589 meaning units that corresponded to the aim of the study were identified, condensed, and coded during the analysis process of the interview texts. The codes were compared based on similarities and differences and grouped into nine sub-themes, which were then compiled into three themes [18]. To enhance transparency, illustrative examples of the analytic process are presented in Table 2.

**Table 2:** Examples of meaning units, condensed meaning units, sub-themes, and themes

Meaning unit	Condensed meaning unit	Sub-theme	Theme
“I think it would have been positive to have a mentor. I really missed some kind of mentoring program or some sort of introduction for new managers” (Participant no. 7)	Lack of mentorship and structured introduction for new managers	Aligning support structures with organizational demands	Providing resilient organizational structures
“To be able to see people who need support and help, it takes so long before you manage to realize, she was supposed to do” (Participant no. 4)	It took a long time to notice who needed support and follow up responsibilities.	Managing time, resources, and workforce capacity	
“To come together and collaboratively to find a solution that is best for us … how we can work” (Participant no. 9)	Together, finding solutions on how to work	Creating unified structures and practices	
“I’m thinking education and networking with other managers a lot… after having righted the ship that was about to capsize, you have to think in new ways .I need to gain knowledge… I’m very much a novice in that area…” (Participant no.1)	Need for education and networking to build knowledge and confidence in the role	Utilising support from leadership and networks	Fostering supportive collaborations
“My fellow manager colleagues are my most important source of support.” (Participant no. 14)	Collegial support from fellow managers is the primary source of leadership support	Maintaining leadership capacity through collegial support	
“We have a lot of support functions at higher levels, but I don’t have any administrative support locally. All scheduling and daily staffing planning rests on me as a manager.” (Participant no.25)	Lack of local administrative support, with full responsibility for scheduling and staffing placed on the manager	Adapting and providing appropriate administrative support	
“Being able to make changes and see that things are improving. It’s so rewarding to see employees develop in their roles and to help and support them. But also to see how they can provide better support to our patients. To make a difference feels meaningful in our area of work.” (Participant no. 20)	To make changes, support employees’ development, improve patient support, and experience a sense of meaning at work	Integrating purpose with everyday leadership	Cultivating leadership for innovation
“Unfortunately, it’s more acceptable to say things publicly to a manager than the other way around. Even if you know it’s about the role, it still affects you, some days you’re more sensitive than others. It’s important not to take things personally” (Participant no.17)	Managers experience emotional exposure and blurred boundaries between role and person, requiring resilience in handling public criticism	Managing complexity without clear boundaries	
“Difficulty in drawing boundaries between being visible and present in the workgroup and having uninterrupted time to complete managerial tasks” (Participant no.11)	Struggling to balance visibility in the team with uninterrupted administrative responsibilities	Balancing leadership with task-focused duties	

The first author (AM) and the last author (IL) performed the analysis, with ongoing discussions within the multidisciplinary research group until a consensus was reached. The research group had extensive experience in caring sciences and qualitative methods, including registered nurses (AM, PS, AG, IL), a midwife (AM), a physician (BA), and a medical scientist (JN).

### Reflexivity

Reflexivity is a central component in qualitative research, involving the researchers’ continuous consideration of how their own experiences, preconceptions, and positions may influence the research process [21]. Reflexivity in this study was integrated by being aware of the researchers’ presence during the interviews and of their influence on the interpretative process. The multidisciplinary research team engaged in reflective discussions on how their professional backgrounds and preunderstandings might influence the way data were understood throughout the study. These reflections guided the analytical work and contributed to transparency and sensitivity in relation to the participants’ narratives.

### Ethics

The study was approved by the Swedish Ethical Review Authority (ref. no. 2023-08149-01) and conducted in accordance with the Declaration of Helsinki [22].The study fulfilled the ethical requirements of research in terms of information, informed consent, confidentiality, and participant safety. All the participants received both oral and written information about the study and provided informed consent prior to participation. They were informed that participation was voluntary and that they could withdraw at any time without giving a reason. The data were anonymised so that the results were presented in a way that prevents individual identification and were only accessible to the research team.

## Results

The findings showed three overarching conditions for sustainable leadership among first-line managers: Providing resilient organizational structures, Fostering supportive collaborations, and Cultivating leadership for innovation. Providing resilient organizational structures described structural conditions, including aligning support structures with organizational demands, managing time, resources, and workforce capacity, and creating unified structures and practices. Fostering supportive collaborations described social conditions, including utilizing support from leadership and professional networks, maintaining leadership capacity through collegial exchange, and ensuring access to appropriate administrative support. Cultivating leadership for innovation described individual conditions related to first-line managers’ everyday leadership practices, including integrating purpose with everyday leadership, managing complexity without clear boundaries, and balancing leadership with task-focused duties. The themes and sub-themes are presented in Table 3.

**Table 3:** Themes and sub-themes describing conditions for sustainable leadership among first-line managers in healthcare settings

Themes	Providing resilient organizational structures	Fostering supportive collaborations	Cultivating leadership for innovation
**Sub-themes**	Aligning support structures with organizational demands	Utilising support from the leadership and the networks	Integrating purpose with everyday leadership
	Managing time, resources, and workforce capacity	Maintaining leadership capacity through collegial support	Managing complexity without clear boundaries
	Creating unified structures and practices	Adapting and providing appropriate administrative support	Balancing leadership with task-focused duties

### Providing resilient organizational structures

Providing resilient organizational structures emerged as a structural condition for sustainable leadership, encompassing three interrelated conditions. Aligning support structures with organizational demands concerned how support functions were experienced as responsive and accessible to managerial needs. Managing time, resources, and workforce capacity concerned ongoing balancing between operational demands and development work. Creating unified structures and practices concerned shared routines, values, and arenas for leadership reflection. Together, these conditions described how organizational structures shaped first-line managers’ opportunities to sustain leadership in everyday practice.

#### Aligning support structures with organizational demands

Aligning support structures with organizational demands was identified as a condition for sustainable leadership. First-line managers described that clear, responsive support functions and systems facilitated navigation of the managerial role. When support functions and systems were unclear, processes often had to be worked out independently. This was described as contributing to a sense of being “thrown into” the managerial role without clear guidance or structure.

A need for a structured induction program tailored to the specific challenges of the role was described to reduce initial uncertainty and provide clearer direction for new managers. The first-line managers suggested that such programs should be accessible (e.g., digitally) and embedded in organizational routines rather than depending on individual prioritization.

“*Maybe there needs to be this introduction for new managers in the health authority* more *often or in a digital version*,* so you can use it to introduce the new manager.” (Participant no. 11)*

It also emerged that participation in existing induction programs often required first-line managers to actively prioritize their own time, which could be difficult in an already demanding work environment. A recurring proposal was to allocate dedicated time for onboarding as a formal part of the transition into the managerial role.

An imbalance was described between internal leadership development programs and recruitment into first-line management positions. Internal programs for aspiring leaders were described as demanding, while appointments to first-line management could occur without formal leadership training before starting the role.

“*No formal leadership training is required when being recruited as a manager—you go directly from being a nurse to being a manager*,* and are trained in leadership after being hired*.” *(Participant no. 6)*

This inconsistency between recruitment standards and the content of leadership development programs was described as a barrier to coherent leadership support. First-line managers described a need for ongoing professional development and improved organizational structures to bridge the gap between expectations and preparation. Continuous leadership training was also described as supporting sustainable leadership, particularly during periods of change and organizational development.

#### Managing time, resources, and workforce capacity

Managing time, resources, and workforce capacity emerged as a challenge in the practice of sustainable leadership and was described as closely tied to shortcomings in existing organizational structures and support systems. First-line managers reported that prioritizing certain tasks often left other responsibilities unaddressed, contributing to a constant sense of inadequacy and blurred boundaries between work and personal life.

*“One works all hours of the day*,* really. Because you try to do everything and arrange everything. I’ve become much better at learning to prioritize*,* and I think that is also part of it*,* if you go back to sustainability; you need to learn to prioritize.” (Participant no. 20)*

Work-related pressures frequently extended beyond official working hours. First-line managers described how problem-solving and responsibilities followed them into their private lives. These circumstances were described as limiting the ability to lead organizational change and development in a sustainable way. Uninterrupted time to manage change processes, foster employee engagement, and enable participation was described as needed.

Access to sufficient resources, in terms of both time and manageable team sizes, was described in relation to maintaining continuity in leadership tasks. Managing large teams was experienced as challenging, as it hindered efforts to build meaningful relationships and provide individual support. This, in turn, risked leaving leadership responsibilities unfulfilled and negatively impacting both the work environment and development efforts.

*“That one has time to carry out the tasks and responsibilities that come with being a manager. That’s probably the hardest part*,* and I think that in this context*,* it becomes important for me that the staff groups aren’t too big*,* meaning too many employees per manager.” (Participant no. 16)*

Overall, team size was described as shaping conditions for sustainable leadership. Difficulties in managing both operational demands and individual staff needs were described as evident in around-the-clock services. Challenges related to staff turnover and recruitment were described as further increasing workloads and stress levels, making it harder to meet operational goals and support employees. These conditions were described as limiting to effective distribution of time and resources, thus constraining opportunities to initiate and lead long-term development within the organization.

#### Creating unified structures and practices

Creating unified structures and practices was described as a condition for fostering a resilient organizational culture that supports sustainable leadership. First-line managers emphasized the need for shared routines and working methods across different units. The absence of clear, unified strategies and insufficient coordination between departments were experienced as barriers to both effective collaboration and well-functioning organizational processes.

Differences in physical conditions across sites were described as complicating the allocation of personnel resources, which in turn affected task distribution and led to workload imbalances. This challenge was further compounded by hierarchical structures and unclear communication channels between workgroups, which were described as barriers to initiating change and implementing common practices.

*“Hierarchical structures between workgroups that make it difficult to reach out to everybody*,* outdated.” (Participant no. 22)*

First-line managers described how a fragmented, siloed approach, in which units operate largely in isolation, led to a loss of overall perspective. This fragmentation was perceived to reduce the hospital’s operational efficiency and limit its collective capacity for development and collaboration. Such organizational conditions were described as constraining not only the leadership’s ability to act during periods of change but also the long-term potential for leading in a sustainable way.

*“Working in silos that cause us to lose the overall perspective.” (Participant no. 2)*

### Fostering supportive collaborations

Fostering supportive collaborations emerged as a social condition for sustainable leadership, encompassing three interrelated conditions. Utilising support from the leadership and the networks concerned how formal leadership structures and informal professional networks enabled first-line managers to fulfil their responsibilities in a complex, changing healthcare environment. Maintaining leadership capacity through collegial support concerned how collegial exchange and shared experiences contributed to sustaining leadership capacity over time. Adapting and providing appropriate administrative support concerned how administrative support created space for leadership and clarified boundaries between managerial responsibilities and support functions. Together, these conditions described how social conditions shaped first-line managers’ opportunities to sustain leadership in everyday practice.

#### Utilising support from the leadership and the networks

Utilising support from leadership and professional networks was described as part of fostering supportive collaborations. First-line managers described the importance of having a responsive and engaged supervisor, someone who recognized efforts, provided constructive feedback, and offered support. Sustainable leadership was described as requiring time for meaningful discussions about interpersonal and emotional aspects, rather than focusing solely on information exchange. Feeling noticed and appreciated by leaders was described as contributing to the work situation, and acknowledgment of workload, recovery, and balance was also described.

Supportive collaborations beyond direct supervision were described as including informal discussion groups, organized leadership networks, and mentorship programs. First-line managers described a need for dedicated forums for joint reflection on leadership and the managerial role, which were often lacking in daily practice.

*“Have little opportunity to talk about leadership in everyday life*,* lack guidance.” (Participant no. 12)*

Interest in mentorship programs was described in relation to navigating managerial responsibilities and complex personnel matters. At the same time, challenges in accessing mentorship were described, including unclear program structures and limited organizational support.

*“There is a mentorship program*,* but it is difficult to find someone to match with; you choose from the system who you want to have as a mentor. And there isn’t a proper guide in the system.” (Participant no. 18)*

#### Maintaining leadership capacity through collegial support

Collegial support was described as contributing to sustained leadership capacity over time, particularly through opportunities to share responsibility, discuss difficult situations, and reflect together. First-line managers experienced that shared leadership structures shaped the everyday conditions for collegial support and fostered reciprocal engagement within managerial teams.

Being part of a shared leadership model characterized by close collegial cooperation was described as distributing responsibility and reducing the burden on individual managers. Opportunities to share workloads and engage in collective reflection on complex situations were described as supporting the managerial role over time. Conversely, the absence of collegial support was described in relation to increased role strain and stress.

*“* *I don’t think I would have stayed so long in that position if I hadn’t been able to exchange ideas and discuss things with my colleague.* *”* *(Participant no. 25)*

Feelings of inadequate support and professional loneliness were described in relation to the leadership role. Addressing such experiences was described as requiring engagement in mutual support, including setting aside reluctance to seek help. First-line managers described pride in the collective work as supporting sustainable leadership, rather than focusing solely on individual performance.

*“You need to feel proud of what you do*,* both individually and together.” (Participant no. 9)*

Non-hierarchical and non-prestigious clinical leadership was described as supporting collegial exchange. Everyday work was experienced as more demanding during periods of organizational change and quality improvement when collegial support was limited. Collegial support was described as enabling first-line managers to navigate challenges, support recovery, and sustain leadership over time.

#### Adapting and providing appropriate administrative support

Adapting and providing appropriate administrative support was described as contributing to sustainable leadership by relieving routine tasks and reducing operational burden. First-line managers described both opportunities and obstacles in administrative functions, while frustration and uncertainty were described in relation to unclear divisions of responsibility and vague support roles.

*“It is necessary to have a clearer division of responsibilities between management and support functions.” (Participant no. 4)*

Although support functions were formally available, first-line managers described difficulties in understanding what assistance was offered and how to initiate contact. Challenges in reaching the right person for specific tasks were described as recurring, leading to delays and inefficiency. A need for clearly defined boundaries between managerial responsibilities and support functions was described in relation to reducing operational burden and creating time for strategic work.

Accessible administrative support was described as facilitating routine tasks, freeing time for leadership activities such as follow-up, staff dialogue, and development work.

*“The hardest part of being a manager is probably the everyday issues that can arise; it can take some time before you get in touch with the right people.” (Participant no. 10)*

At the same time, the role of support functions was described as unclear, which hindered collaboration. First-line managers requested functions with clearly defined authority and responsibility and described that support inquiries sometimes resulted in counter-questions rather than concrete assistance. Organizational centralization was described as transferring more tasks to managers, reducing first-line managers’ access to support staff, and being present in daily operations.

*“Everything has been centralized*,* which means that everything has been placed on the manager instead.” (Participant no. 17)*

The implementation of new systems, such as digital scheduling tools, was described as adding to the workload. Expectations that first-line managers would handle the entire scheduling process were described as consuming time needed for other leadership responsibilities, including initiatives for change. A need for additional administrative support was described in relation to managing routine tasks and creating space for leadership with a long-term, sustainable focus.

### Cultivating leadership for innovation

Cultivating leadership for innovation emerged as an individual condition for sustainable leadership, encompassing three interrelated conditions. Integrating purpose with everyday leadership concerned how meaning was described in relation to managerial roles and everyday leadership work. Managing complexity without clear boundaries concerned how evolving responsibilities and ambiguous expectations were described as shaping the managerial role. Balancing leadership with task-focused duties concerned how leadership presence and communication with staff were balanced in relation to administrative demands and competing priorities. Together, these conditions described how leadership was shaped by individual conditions in everyday practice.

#### Integrating purpose with everyday leadership

Integrating purpose with everyday leadership was described as a part of cultivating leadership for innovation. First-line managers described purpose in relation to contributing to development efforts and balancing operational tasks with leadership responsibilities. Prioritizing, adapting to change, and acting independently and collaboratively were described as part of managing everyday leadership demands. This was described to manage leadership complexity, involving both task focus and relationship building. First-line managers described that sustaining leadership over time depended on having autonomy to plan their workday and access to support when needed.

“*The right conditions are when I feel that I can do a good job both for the group and for the organization.” (Participant no. 7)*

The ability to plan the workday, allow time for recovery, and take breaks without frequent interruptions was described as related to remaining in the role over time. Integrating the managerial role with a balanced personal life was described as supporting sustainable leadership over time.

“*To be a manager provides the freedom to plan your work and manage your time.” (Participant no. 5)*

#### Managing complexity without clear boundaries

Managing complexity without clear boundaries was described as a part of cultivating leadership for innovation. Lack of clarity regarding the full scope of the managerial role was described, with the extent of responsibilities becoming apparent after taking on the position. A need for transparency during the recruitment process was described, including communication of existing challenges and organizational goals, to prepare managers for the complexities of the role.

*“It is important to have clarity about the assignment during the interview for the position*,* specifying the problems that exist and what one hopes to achieve with the department moving forward.” (Participant no. 13)*

First-line managers described a need for leadership structures that ensured appropriate competencies and clarified the purpose and responsibilities of the managerial role. Tasks were described as evolving over time, with increased demands to participate in processes of change and to contribute to strategic development. First-line managers described needing time to understand the role and make decisions with confidence. Reflections were provided on whether the timing in life was suitable for assuming a managerial position, and, in some cases, on the consideration of leaving the role. Motivation to perform at a high level was described, alongside experiences of inadequacy when demands exceeded available time. The pace of work was described as contributing to maintaining a façade of control while experiencing a sense of falling short. Frequent managerial changes in the first-line function were described as affecting continuity and day-to-day operations.

*“One can walk around with a feeling of failure*,* thinking that one could have performed better*,* and a sense of inadequacy when one doesn’t have time to prepare for meetings/projects as desired” (Participant no. 4)*

The ability to manage complex tasks was described as dependent on individual strategies, such as working in a structured manner and drawing on prior experience. First-line managers described relying on their own ability to navigate uncertainty in the absence of organizational support.

#### Balancing leadership with task-focused duties

Balancing leadership with task-focused duties was described as a part of cultivating leadership for innovation. First-line managers described challenges reaching out to and communicating with employees, as well as difficulties being consistently present during daily activities. Expectations of greater managerial presence were described in relation to employees’ needs, while limited opportunity for presence was described in relation to competing demands. Misunderstandings about roles and expectations were described as contributing to tensions in the work environment, including criticism directed at the first-line manager and expressed in a cynical tone, for example, through emails.

*“Employees and managers need to become aware of each other’s work in some way.” (Participant no. 19)*

Difficulties in setting boundaries between being visible within the workgroup and having uninterrupted time for focused work were described. First-line managers described the need to be present to lead and guide daily activities, while administrative demands were described as competing with this requirement, particularly in larger organizations. At the same time, support from employees was also described, including shared responsibility and collaborative engagement to address challenges.

*“And that the employees also take part in sharing the responsibility to help achieve structure.“(Participant no. 1)*.

This engagement was described as involving shared responsibility for establishing clear structures and well-organized processes within the department, which facilitated the first-line manager’s work and supported collaboration. Setting boundaries and shared responsibility were described in relation to sustaining leadership over time.

## Discussion

The findings indicated that sustainable leadership among first-line managers in healthcare is influenced by the interaction between structural, social, and individual conditions. Structural conditions involved providing resilient organizational structures and were characterised by aligned support functions that met operational requirements, resource allocation, and coordination of work routines and practices. These aspects contribute to the ability to carry out leadership tasks over time. Social conditions involved fostering supportive collaborations and comprised supervisory and peer support, opportunities for collegial exchange, and access to administrative assistance. These forms of support affected how managers handled their responsibilities and responded to organizational change. Individual conditions involved cultivating leadership for innovation and included connecting leadership tasks to a clear purpose, managing shifting expectations, and balancing operational duties with broader development goals. Together, these findings describe the various conditions that shape first-line managers’ opportunities to fulfil their leadership roles. While some conditions were primarily organizational and often beyond first-line managers’ direct control, others were shaped by collegial relations and first-line managers’ everyday leadership practices.

The findings at the structural level showed that sustainable leadership depends on organizing activities within clear frameworks supported by resources that align with both organizational demands and real-world conditions. This entails adapting support systems, balancing time and staffing resources, and nurturing a cohesive organizational culture. These factors interact to create a foundation that enables managers to perform their duties sustainably. However, unclear role expectations, especially during onboarding and induction, are a recurring issue that risks weakening managers’ ability to lead over time [10, 11]. Addressing this requires mandatory induction programs with explicit role expectations, strengthened administrative support functions, and clearer role definitions, all of which can reduce administrative burdens and allow increased presence in clinical work [23]. In addition, the findings in this study showed that ambiguous delegation boundaries increased the managers’ workload and complicated effective task distribution. Taken together, this points to the fact that sustainable leadership requires the organization to establish structural conditions that not only provide resources but also clarity and balance, which are crucial for managers’ long-term capacity.

At the social level, collegial support and professional networks emerged as core resources that enabled managers to cope with strain and organizational changes. Trustful collaborations and shared responsibility within the leadership team created a sense of security and engagement and served as a buffer during periods of organizational pressure. This interpretation is supported by previous research, where trust, collegial support, and collaboration within the management team were described as important for remaining in the role and sustaining commitment over time [5], while lack of support was shown to intensify strain and contribute to leaving the role [4]. Such social resources have also been linked to empowerment and managers’ well-being [24, 25]. At the same time, our study extends this understanding by underscoring the importance of arenas for reflection and exchange of experience, especially during processes of change where loneliness and responsibility can become especially burdensome. Consequently, social resources need to be formally organized rather than left to informal networks alone. A cohesive and supportive culture further facilitates managerial work and contributes to improved care quality by reducing silo structures and promoting interprofessional collaboration [26, 27]. Breaking down hierarchical barriers and creating flexible organizational structures for resource allocation, appropriately sized teams, and time planning, are likewise critical for balancing developmental work with daily staff management. Overall, this shows that social support and an inclusive culture act as vital buffers and resources for sustainable leadership. Furthermore, clear communication, participation, and relational competence are essential factors that reinforce these social conditions and have been identified as central in sustaining leadership [17, 28].

At the individual level, our findings underscore the importance of a manager’s ability to create meaning in their leadership, act with integrity, and be flexible in the face of complex and changing conditions. This is crucial to maintaining motivation and energy. Such reflective and flexible leadership, in which managers are given both the freedom to act and support, has been identified as a protective factor against burnout [13, 17]. Our study also show that first-line managers often experience exclusion from strategic decision-making, which leads to reduced participation and limits their operational expertise. This corresponds with previous research demonstrating that a lack of managerial involvement in strategic processes can diminish both their motivation and the organization’s capacity to achieve desired outcomes [29, 30]. This may be understood as a structural limitation, in which practical insights and experiences from managerial levels are overlooked in decision-making processes, potentially resulting in decisions less aligned with the realities of the organization and complicating implementation [4, 23, 31]. The present study shows that the possibility for managers to plan their own workday, create space for recovery, and take time off without continuous work-related interruptions contributed to their ability to remain in their role over time. The ability to integrate the managerial role with a well-functioning personal life was described as a necessary condition for exercising committed and sustainable leadership in the long term. Organizational structures that not only support managers’ roles but also enable their influence and participation are important for leadership [15]. Furthermore, the balance between demands and resources affects managers’ motivation and job satisfaction. Workloads, clear mandates, and the ability to delegate tasks are factors related to stress and burnout among first-line managers [11, 14, 32]. While structural conditions shape managerial participation, sustainability at the individual level also depends on reflective, flexible, and future-oriented leadership capacities, as illustrated in our findings and supported by previous research [15, 33]. Such capacities enable adaptation to the complex and changing conditions characteristic of the healthcare sector [15, 33]. Taken together, the findings extend previous leadership research in healthcare, which has primarily focused on leadership styles and their influence on employee engagement [8]. In contrast, this study conceptualizes sustainable leadership as the multi-level conditions that enable first-line managers to maintain leadership capacity over time, and it specifies the structural, social, and individual mechanisms through which this capacity is sustained. This shifts the understanding of sustainable leadership from an individual leadership quality to a contextually embedded and relationally produced capacity. By clarifying how these mechanisms interact in practice, the study offers a more comprehensive understanding of how sustainable leadership is developed and sustained in complex healthcare environments [29, 30].

For practice, the results highlight the importance of developing stable organizational structures and adequate resources, while also establishing formal social arenas to support collegial trust, shared responsibility, and reflective dialogue. Leadership development should focus on promoting flexibility, self-reflection, and the ability to create meaning in complex tasks. Together, these integrated efforts can contribute to resilience at both individual and organizational levels.

### Strengths and Limitations

Trustworthiness in qualitative research is often assessed based on five criteria: credibility, dependability, confirmability, transferability, and authenticity. Credibility was strengthened by allowing participants to share their experiences freely through open and non-leading interview questions. The researchers were conscious of their own presence and influence during the interviews and ensured that both physical and digital formats were used based on participants’ preferences, thereby acknowledging individual needs and comfort levels [18, 19, 21]. These strategies helped ensure that participants’ perspectives were accurately captured and reflected in the results.

To further enhance the dependability, the researchers followed the COREQ checklist, which supports transparency and rigor in qualitative research. The analysis process was documented and conducted collaboratively within a multidisciplinary research group, ensuring a systematic and coherent approach throughout the study [18, 19, 21]. The research team consisted of the PhD student and four supervisors. Three supervisors were nurses from different healthcare specialties, two professors, and one PhD, while the fourth was a physician with a PhD. This diverse composition added breadth and depth to the interpretation of the data and contributed to the methodological stability of the research.

Confirmability was addressed through a reflexive approach maintained throughout all phases of the study. The researchers continuously reflected on their own preconceptions and engaged in critical dialogue within the research group to minimize bias and maintain objectivity in the analysis. Previous research emphasizes the importance of reflexivity in qualitative research, underscoring the need for researchers to be aware of and actively reflect on their own perspectives [18, 19, 21]. The two primary researchers (AM and IL) in this study worked closely with the multidisciplinary team to ensure that interpretations were well-founded and grounded in the data rather than in the researchers’ assumptions.

Transferability was also considered in the study design and analysis. The sample consisted primarily of women (23 out of 25 participants), which may have influenced the perspectives expressed, particularly regarding leadership and working conditions. The researchers reflected on how this gender distribution might have shaped the results and acknowledged that a more diverse sample could have offered a broader range of experiences [18, 19, 21]. The sample also included first-line managers from different hospital departments and with variation in managerial experience, which supported breadth in the accounts. Although participants were recruited from three different hospitals, all were located within the same health authority, which may limit the transferability of the findings to other organizational contexts. Seventeen of the 137 invited participated, 5 declined, and 115 did not respond. The low response rate, possibly due to time constraints, lack of interest, or work-related barriers, should be acknowledged as a limitation when interpreting the findings. By providing detailed descriptions of the context, participants, and data collection process, the study offers sufficient information for readers to assess the transferability relevance of the findings to other settings. As the study focused on first-line managers’ experiences, other perspectives (e.g., staff, senior management, or other stakeholders) were not included. This may influence how the findings transfer across organizational levels.

Finally, authenticity was a guiding principle throughout the research process. The goal was to represent the participants’ experiences in a balanced and meaningful way. By offering flexible interview formats and creating space for genuine, uninfluenced expression, the researchers ensured that participants’ voices were faithfully represented. The study aimed to give visibility to a range of perspectives, thus ensuring that the findings present a rich and faithful account of the participants’ realities.

## Conclusion

This study shows that sustainable leadership among first-line managers in healthcare develops from the dynamic interaction of structural, social, and individual conditions. Rather than being attributable to single factors, sustainable leadership develops when organizational systems are coherent, relational support is accessible, and managers can integrate purpose and adaptability into their leadership practice.

These insights emphasise that strengthening sustainable leadership requires strategies that address organizational prerequisites, collaborative processes, and individual capacities simultaneously. The findings may inform the design of initiatives that support first-line managers in demanding contexts, including leadership development efforts that promote collegial learning, well-being, and preparedness for managing change. Future research may further explore how first-line managers lead development processes in healthcare, including perspectives from staff and senior management, particularly in relation to emerging demands such as digitalisation and the implementation of advanced technologies.

## Supplementary Information

Below is the link to the electronic supplementary material.

Supplementary Material 1

## Data Availability

The datasets generated during and/or analyzed during the current study are not publicly available due to legal and ethical restrictions surrounding participant confidentiality. De-identified excerpts of the data can be made available from the corresponding author at a reasonable request. Requests for data access may be subject to approval by the relevant ethics committee. For further information, please contact the corresponding author.
