# Supplementary material for: Exploring sustainable leadership among first-line managers in healthcare: a qualitative study
Source: BMC Health Serv Res. 2026 Mar 23;26:491. doi: 10.1186/s12913-026-14412-6 (PMC13063711; doi:10.1186/s12913-026-14412-6)
Supplement: Supplementary file 1 — Supplementary Material 1 [file 12913_2026_14412_MOESM1_ESM.pdf]

Interview guide

Introductory questions

- Tell me about your background and education.
- Tell me about your work as a first-line manager.

Main questions

- What does sustainable leadership as a manager mean to you, and how would you describe it?
- How do you, in your role as a first-line manager, work to promote a sustainable working life in practice?
- Which opportunities do you have to achieve a sustainable working life, and how do you experience them?
- Which obstacles do you see in achieving a sustainable working life, and how do you perceive them?

Questions related to long-term sustainable working life

- What makes your work as a first-line manager sustainable over time?
- What helps you cope with your workload in a way that is sustainable in the long run?
- What threatens the long-term sustainability of your working life as a manager?

Probing questions (used throughout the interview as needed)

- Can you tell me more about that?
- What do you mean by that?
- Can you describe that further?
- What did you have in mind when you said that?
- Can you give an example?
- How did that affect you or your work situation?
